# Feasibility of Machine Learning Algorithms for Predicting the Deformation of Anodic Titanium Films by Modulating Anodization Processes

**DOI:** 10.3390/ma14051089

**Published:** 2021-02-26

**Authors:** Sung-Hee Kim, Chanyoung Jeong

**Affiliations:** 1 Department of Industrial ICT Engineering, Dong-eui University, 176 Eomgwang-ro, Busanjin-gu, Busan 47340, Korea; sh.kim@deu.ac.kr; 2 Department of Advanced Materials Engineering, Dong-eui University, 176 Eomgwang-ro, Busanjin-gu, Busan 47340, Korea

**Keywords:** titanium oxide, systematic surface control, machine learning, nanostructure prediction, anodization

## Abstract

This study aims to demonstrate the feasibility of applying eight machine learning algorithms to predict the classification of the surface characteristics of titanium oxide (*TiO*_2_) nanostructures with different anodization processes. We produced a total of 100 samples, and we assessed changes in *TiO*_2_ nanostructures’ thicknesses by performing anodization. We successfully grew *TiO*_2_ films with different thicknesses by one-step anodization in ethylene glycol containing *NH*_4_*F* and *H*_2_*O* at applied voltage differences ranging from 10 V to 100 V at various anodization durations. We found that the thicknesses of *TiO*_2_ nanostructures are dependent on anodization voltages under time differences. Therefore, we tested the feasibility of applying machine learning algorithms to predict the deformation of *TiO*_2_. As the characteristics of *TiO*_2_ changed based on the different experimental conditions, we classified its surface pore structure into two categories and four groups. For the classification based on granularity, we assessed layer creation, roughness, pore creation, and pore height. We applied eight machine learning techniques to predict classification for binary and multiclass classification. For binary classification, random forest and gradient boosting algorithm had relatively high performance. However, all eight algorithms had scores higher than 0.93, which signifies high prediction on estimating the presence of pore. In contrast, decision tree and three ensemble methods had a relatively higher performance for multiclass classification, with an accuracy rate greater than 0.79. The weakest algorithm used was k-nearest neighbors for both binary and multiclass classifications. We believe that these results show that we can apply machine learning techniques to predict surface quality improvement, leading to smart manufacturing technology to better control color appearance, super-hydrophobicity, super-hydrophilicity or batter efficiency.

## 1. Introduction

Nowadays, ordered titanium dioxide (*TiO*_2_) nanotube arrays obtained by *Ti* anodization have overwhelmingly attracted scientific and technological interests because of their functional properties. Among its various applications, *TiO*_2_ plays a pivotal role because of its chemical stability, nontoxicity, and biocompatibility [1,2,3]. For example, *TiO*_2_ is used in solar cells production [3], photocatalytic processes [4], and self-cleaning coatings [2,5]. It also has antibacterials properties [6] and can be used in semiconductors [2]. *TiO*_2_ is also considered a synthetic bone graft substitute [7,8]. Considering their superior features including unique structure, high specific surface area, and quantum confinement effect, *TiO*_2_ nanotubes/nanoporous arrays are the most frequently fabricated nanostructures. Therefore, various methods such as template-assisted sol-gel synthesis, play a crucial role in the fabrication of *Ti* nanostructures [9,10]. However, highly aligned nanotubes/nonporous structures have increasingly focused on the anodization of *Ti*. Anodization is a simple electrochemical process performed to produce thick nanoporous/tubular metal oxide and subsequently precious such as *Al*, *Ti*, and *Mg* [11,12]. In general, the majority of principles that apply to the electrochemical anodization of *Ti* in electrolytes containing fluoride ions is the most common method used to create self-organized nanotube arrays [13,14,15]. The anodic formation of *TiO*_2_ is associated with different parameters, which affect the surface morphology and quality of the structure [16,17,18]. For example, by controlling anodization parameters, such as electrolyte type, electrolyte composition, pH, applied voltage and potential difference, temperature and anodization duration, nanostructures with different morphologies and characteristics can be obtained [14,15,19,20]. However, combining all these parameters during anodization to observe all types of surface characteristics is costly. It would be helpful if we can narrow the experimental procedures to a certain range by predicting the core surface factors, which we aim to determine in our present study. Herein, we focused to fabricate nanoporous/nanotubes anodic titanium oxide layers through a one-step anodization process performed in fluoride-containing ethylene glycol at various anodization and voltages. We mainly investigate the influence of applied voltage and duration difference on the growth of *TiO*_2_ nanostructures. Based on the systematic control over the two parameters, we obtained 100 samples and categorized surface characteristics from the surface image. We investigated whether we could apply machine learning algorithms to predict an anodic formation of *TiO*_2_ structure. We clearly aimed to predict surface characteristics that were classified into two or four groups based on pore structures. Besides the common parameters assessed in *TiO*_2_ structures such as surface thickness, these oxide structures are considered significant because they are superhydrophilic, superhydrophbic, and have excellent color appearance on a *Ti* surface. We believe that if we are able to determine certain parameters of the pore structures without conducting labor-intensive experiments, the time and cost of conducting several experiments can be sufficiently utilized. In this study, a total of 100 samples were produced, and this sample size is relatively small when assessing the feasibility of machine learning algorithms compared to other domains. However, if we can determine the feasibility of predicting surface characteristics, we will be able to harness the appropriate predictive model that should be used to estimate generated surface characteristics, allowing us to focus more on higher granularity of experimental conditions to generate the desired surface characteristics.

Machine learning is a type of artificial intelligence algorithm that uncovers patterns in large datasets using computer-based statistical models. Recently, machine learning has developed a wide range of algorithms, which can be roughly described in three ways: (1) supervised learning, where input and output variables are given and the model is determined from the labeled input dataset; (2) unsupervised learning where a self-organized learning method determines unknown patterns in a dataset without pre-existing labels; and (3) reinforcement learning, where an intelligent agent is defined and interacts with its environment by performing actions and learning from errors or rewards, termed as a trial-and-error approach for learning [21].

Classification is a type of supervised learning technique that categorizes data from prior information. The algorithm is used to predict a discrete value that is assigned to a particular class or group. Imagine a set of photographs of animals where each photo is labeled as a cat, rabbit or other animals. When a new image is assigned, the algorithm has to classify it into one of these labeled categories. Each testing instance is matched with a category, which we call labeled data and is used for training. Classification is performed in two phases, and the labeled dataset is divided into training and test dataset. First, a classification algorithm updates its model with the training dataset, and the analytical model extracted is validated against a labeled test dataset to calculate the model performance and accuracy. Compared to different applications such medical imaging detection or quality control in manufacturing, materials science has recently applied these machine learning techniques considering the difficulties they faced in collecting a large sample of data. However, the prerequisite for machine learning is the existence of prior data. To obtain thousands of input data with traditional experimentation methods, a significant amount of cost, time, and effort is required in materials science. Therefore, current research in materials science primarily aims to discover and design new materials by using public datasets on material properties [22]. In our current study, we aim to evaluate and compare eight machine-learning techniques in predicting the experimental result on *TiO*_2_ structures, such as pore structure and thickness, by performing different anodization processes.

## 2. Experimental Dataset Development

We degreased the *Ti* sheets by sonicating in a solution of acetone, ethanol, and deionized (DI) water for 30 min. Subsequently, we applied two different polishing methods. Electrochemical polishing was performed in a mixture containing acetic acid, sulfuric acid and hydrofluoric acid (60:15:25 in volume) at a constant current density of 140 mA/cm and a temperature of 20 °C for 1 min. Chemical polishing was performed by dipping *Ti* samples into a stirred mixture of hydrofluoric acid and nitric acid (1:3 in volume) for 10 s. Subsequently, the samples were rinsed with water and ethanol and dried in the air. A combined pretreatment method was conducted by electrochemical polishing followed by chemical polishing. The polished *Ti* samples were prepared via one-step anodization in an electrolyte of ethylene glycol containing 0.25 wt% *NH*_4_*F* and 2 wt% DI water at 0 °C. The process was performed at 10 V intervals from 10 V to 100 V in a two-electrode cell, with polished *Ti* samples as anodes and platinum as a cathode. The duration of anodization was at 1 min intervals from 1 min to 10 min. Structural and morphological characterizations were executed using a field emission scanning electron microscope (FE-SEM). The structural features and thickness of anodized samples were examined directly from FE-SEM images by Image J software.

## 3. Machine Learning Algorithm Development

### 3.1. Data Preprocessing for Classification

The data were categorized based on the characteristics of surface morphology. We introduced the methodology for the multiclass and binary class categorization on the obtained data. Initially, the surface was labeled into four groups based on the images obtained from FE-SEM images. The second author designed the coding scheme based on the formation of an additional layer, roughness with the hexagonal pattern and pore formation. Each aspect is built upon another, for example, a pore would be created after a layer was formed. Both authors used the scheme and independently coded the same 100 images. The inter-rater reliability was found to be good with kappa = 0.94. The two raters slightly modified the coding scheme after validation. The four classes are described below (see Table 1 for examples):

- Class 0: oxide layer creation
- Class 1: oxide layer creation with roughness
- Class 2: oxide layer creation with pore creation
- Class 3: oxide layer creation with uniform pore generation

After categorizing the four classes, the images were classified into two categories, according to the presence of the pore structure: “with pore” and “without pore.” *Without pore* category includes Classes 0 and 1, and *with pore* category includes Classes 2 and 3. A categorization into four classes is advantageous because it results in more granularity; however, because of the small dataset size, we also wanted to test binary classification. The presence of the pore is actually the key aspect we aim to determine considering that when the structure becomes uniform with certain height, it contributes to certain characteristics such as color appearance, super-hydrophobicity or super-hydrophilicity.

### 3.2. Classification Algorithms

We performed the classification task with eight well-known machine learning algorithms. We aimed to compare the prediction capability of eight algorithms, namely, logistic regression (LogReg) [23], naïve Bayes (NB) [24], k-nearest neighbors (KNN) [25], support vector machines (SVM) [26], random forest (RF) [27], bagging, gradient boosting tree (GBT) [28,29], and decision tree (DecTree) [30]. Here the algorithms and importance of their comparison in this study were briefly explained [31]. *LogReg* has been widely used for decades even before the advancement of machine learning. It is one type of regression analysis that is conducted when the dependent variable is dichotomous. NB is a simple classification algorithm calculating the conditional probabilities of each input value given in each class value. It is based on strong assumptions about the independence of each input variable. However, it is shown to be effective in many problems. The *KNN* algorithm is a simple and lightweight supervised machine learning algorithm and it assumes that similar things exist close to one another. It is a nonparametric approach and is stable and robust for small sets and lower dimensional data. The *SVM* algorithm is used to determine a hyperplane in an N-dimensional space and robustly classifies the data points. It shows great performance when the number of features is less. The *DecTree* algorithm is used to create a training model that can be used to make decisions on the class by learning simple decision rules inferred from prior data. Decision trees classify the data by starting from the root and compare down to the next leaf/terminal node, with the leaf/terminal node providing the classification of the new data. For multiclass classification problem, the problem can be solved by naturally extending the binary classification techniques when applying the above algorithms [32]. A machine learning paradigm named ensemble learning is widely used where multiple models often called “weak learners”, are trained to solve a certain problem and combined to achieve better results. The main hypothesis of ensemble learning is that combining weak learners can obtain more robust and accurate models. RF, bagging and GBT are all ensemble methods. *RF* is an example of ensemble learning and the logic is simple but powerful if high nonlinearity and complex relationship between dependent and independent variables are observed. *Bagging* enables the weak learners to learn independently and combines them following deterministic averaging processes. *GBT* trains many models in an additive manner and is based on the intuition that when the next model is combined with the previous models, the overall prediction error is minimized.

### 3.3. Performance Measures for Machine Learning Algorithms

#### 3.3.1. Binary Classification

To calculate the performance of the machine learning models, we used K-fold cross-validation because it is suitable for a dataset with limited sizes [33]. Cross-validation is widely used when we have limited data samples and is a resampling procedure to evaluate machine learning algorithms. To obtain the classification accuracy which is a common practice, 10-fold cross validation was used [34]. It is largely applied because it results in a less biased estimate than simply splitting the dataset into train and test groups. The core idea is to shuffle the dataset randomly; separate a subset of data for validation and use the rest to train a model; and use the left-out data to predict. This process is repeated several times and leaves out a different subset of the data for validation until all the given data is used for learning.

For binary classification, we used area under the receiver operating characteristic (ROC) curve (AUC) [35]. We will first introduce other common measures. For a binary classification problem, accuracy is the proportion of correctly classified instances; however, it is a poor measure for an imbalanced dataset (where *TP* = true positive, *TN* = true negatives, *FP* = false positives, and *FN* = false negatives.)

(1) $$Accuracy=\frac{TP+TN}{TP+TN+FP+FN}$$

Precision and recall are additional ways to assess the results by breaking down the accuracy formula. Precision quantifies the number of classes predicted positive that actually belong to the positive class. Precision is a good performance measure to apply when the costs of false positive are high.

(2) $$Precision=\frac{TP}{TP+FP}$$

Recall quantifies how many of the actual positives our model captures through classifying it as positive (true positive). It is a good performance measure when there is a high cost associated with false negative.

(3) $$Recall=\frac{TP}{TP+FN}$$

*F*1 score balances both precision and recall in one number. It is the weighted average of precision and recall. Therefore, this score concerns both false positive and false negative into account. *F*1 score is usually more useful than accuracy, especially when you have an uneven class distribution.

(4) $$F1=2\times \frac{Precision\times Recall}{Precision+Recall}$$

ROC curves shows the trade-off between the true positive rate (synonym for recall) and false positive rate for the extracted model using different probability thresholds. If we lower the classification threshold, the model classifies more items as positive, thus increasing both false positives and true positives. AUC stands for “area under the ROC curve.” The AUC measures the entire two-dimensional area below the ROC curve from (0,0) to (1,1), which means that AUC provides an indication of how good or bad our classifier is performing across all possible classification thresholds. Therefore, AUC ranges in value from 0 to 1. If the classifier is perfect, then the AUC score is 1.0. If the predictions are 100% wrong, then the AUC is 0. The advantage of applying the AUC measure is that it is invariant of data imbalance [36]. For these reasons, we used AUC to estimate the performance of our experiments.

We used the AUC value as the performance measure. A high AUC value greater than 0.8 denotes a reasonably good prediction rate [37], while an AUC value of 0.5 denotes the predictability of a purely random guess such as flipping a coin.

#### 3.3.2. Multiclass Classification

When the number of classes is high, it becomes more complicated to measure the performance. The combinations of true and false classifications for each class increases. Therefore, micro- and macro-averages are calculated for precision and recalls [38]. However, their interpretation differs as these micro- and macro-averages compute slightly different things. In a multi-class classification problem, micro-averaged precision and recall equations are as below where *c* is the class label.

(5) $$MicroPrecision=\frac{\sum_{c}TP_{c}}{\sum_{c}TP_{c}+\sum_{c}FP_{c}}$$

(6) $$MicroRecall=\frac{\sum_{c}TP_{c}}{\sum_{c}TP_{c}+\sum_{c}FN_{c}}$$

In multi-class classification, the count of all false instances is as below (Equation (7)), therefore micro-precision and micro-recall are the same. In other words, every single false prediction will be a false positive for a certain class, and every single negative prediction will be a false negative for a certain class. As accuracy is a harmony mean of precision and recall, we report the accuracy.

(7) $$\sum_{c}FP_{c}=\sum_{c}FN_{c}$$

A macro-average computes the metric independently for each class and then calculates the average by treating all classes equally. On the other hand, a micro-average computes the average aggregating the contributions of all classes. If there is class imbalance, macro average will have lower values. However, it actually indicates the overall accuracy for all classes, that is, predicting each class is equally important; therefore, we also report the macro-average values.

## 4. Results

### 4.1. Experiment Results

Nanoporous anodic *Ti* oxide layers are fabricated by a one-step anodization process. In the present study, it is evident that the dimension of the nanostructure, such as thickness, strongly depends on the anodization conditions used. The key factors are the anodization time and voltage. After some preliminary experiments, considering duration and voltage, the formation of the nanostructure is possible in a mixture of ethylene golycol/DI water/*NH*_4_*F* in a duration ranging from 10 V to 100 V and a duration ranging from 10 min to 100 min. The association between the voltage and time on the thickness of the *TiO*_2_ films formed on polished *Ti* samples was observed by FE-SEM. Table 2A–J shows the surface of *TiO*_2_ produced by anodization at different voltages and times. Table 2A–J shows three types of structures—a non-nanoporous structure on polished titanium, a nanoporous structure, and a nanotubular structure, where structure shape increases with anodization voltage and time. Initially, the films are significantly thin with the non-porous layer. The films produced with the lowest voltage (up to 50 V) are not ordered, comprising small pores or others without pores. The above mentioned case is not observed, however, in the anodization process under a relatively lower duration (1 or 2 min). The formation of pores is not created and irregular. The formation of pores is not evident at an anodization duration of 1 min, and the nanoporous structure at an anodization duration of 2 min could be seen above the applied voltage of 90 V as shown in Table 2A,B. From the results presented in Table 2F–J, when the higher voltage and duration (greater than 50 V and 5 min) of anodization are used, the *TiO*_2_ structures have significant *TiO*_2_ nanotube and display more uniform films. As can be seen, with increasing anodization time and applied voltage, the rate of oxide growth increases and the *TiO*_2_ films become thicker. As anodization of *Ti* procedure is performed, metal ions (*Ti*^4+^) migrate from metal to the oxide/metal interface and dissolve into the solution. At the same time, the oxygen-containing ions (*O*^2−^) are generated at the oxide/electrolyte interface by the field-enhanced dissolution of *H*_2_*O* or *OH*^−^ under the influence of the electric strength (reactions of 8 and 9) [39].

(8) $$Ti\to Ti^{4+}+4e^{-}$$

(9) $$Ti^{4+}+2H_{2}O\to TiO_{2}+4H^{+}$$

**Figure 1:**
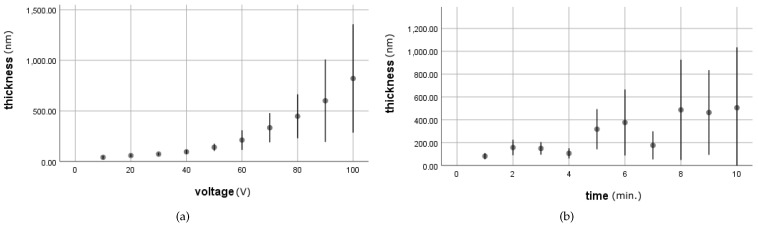
(**a**) Mean thickness by voltage from 10 V to 100 V (**b**) Mean thickness by time from 1 min to 10 min (line indicates 95% CI).

It is clear that the thickness of the grown oxide is closely related to the operating condition during anodization. The effective current efficiency of the formation of *TiO*_2_ in ethylene glycol-based electrolyte is nearly 100%. We can expect that the thickness of the grown oxide is proportional to the electric charge exchanged from electrolyte during anodization process. In other words, the anodizing potential difference by changing current density, anodizing time, and applied voltage determines the thickness of *TiO*_2_. Figure 1 shows the results of thickness for *Ti* anodization in ethylene glycol-based electrolyte. As can be expected, significantly thicker oxide films are created at higher anodizing duration and applied voltage. At relatively higher voltages (greater than 50 V), however, it can be observed that the growth of thickness decreases at specific anodization times. It was considered that the decreasing electrolyte viscosity on the rate of oxide growth in the diffusion during anodization performed in ethylene glycol-based electrolyte. The steady-state oxide growth is a dynamic equilibrium between the rate of oxide growth and the rate of oxide etching at the oxide bottoms. During the oxide growth (which formed metal ions), pH at the oxide bottoms decreases due to reaction 10 [40].

(10) $$Ti+2H_{2}O\to TiO_{2}+4H^{+}+4e^{-}$$

In contrast, decreasing pH at the oxide bottoms encourages the etching of oxide at the oxide film in the ethylene glycol-based electrolyte containing *H*_2_*O* and *NH*_4_*F* (reaction 11) [41].

(11) $$TiO_{2}+6HF\to {\left[TiF_{6}\right]}^{2-}+2H_{2}O+2H^{+}$$

Moreover, it can be attributed to the effect of enhanced hydrolysis of metal ions (*Ti*^4+^) that appear in the electrolyte as a result of the increasing rate of the oxide growth at higher voltage and time. The hydrolysis consequence in the deposition of the hydrous *Ti* oxide on the *TiO*_2_ surface. Consequently, the thickness is reduced by the deposition. Therefore, the rate of deposition not only the hydrolysis is greater than the rate of chemical etching of the *TiO*_2_ layer.

### 4.2. Change of Thickness

**Figure 2:**
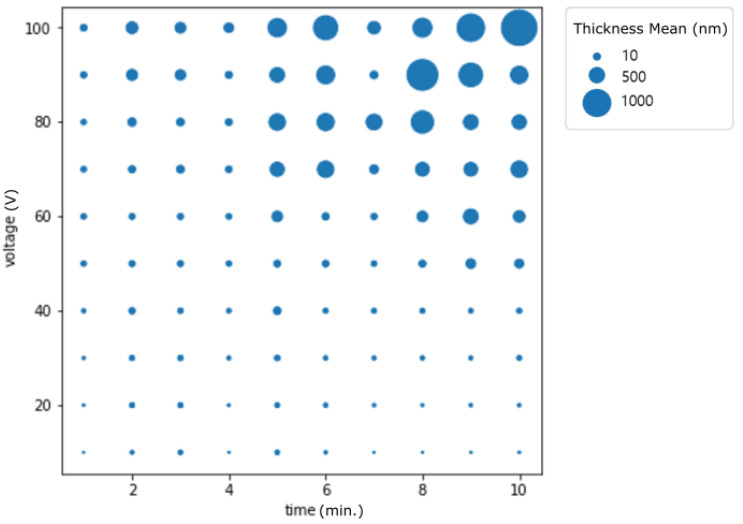
Mean thickness change by voltage and time variation.

Figure 1 shows the mean and variation of thickness by voltage and time separately. Overall, as the voltage and time increase, the mean thickness shows a trend to increase. However, the variation also increases in a nonlinear manner. Compared to voltage change, the fluctuation of the thickness is larger with the time factor. Based on these results, time is a more important factor for oxide thickness change than voltage. Therefore, experiments have shown that time regulation of anode oxidation is a more important factor. Therefore, predicting the anode oxidation time range to obtain the oxide thickness targeted by artificial intelligence can save process time. The following section contains visualizations to help understand the experimental results and to visually inspect how the experimental settings affect the surface and also the relationship by labels that were used for classification. Figure 2 plots the mean thickness by each level of time and voltage that were used in the experiment. The size of the circle indicates the thickness mean.

**Figure 3:**
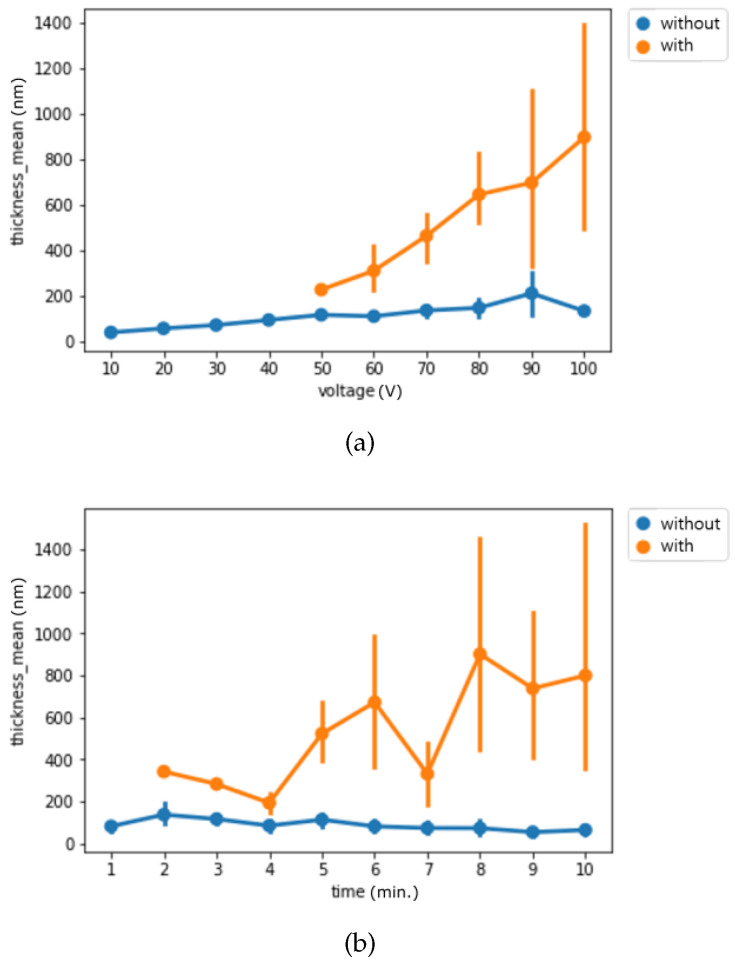
(**a**) Binary classification result by voltage from 10 V to 100 V (**b**) Binary classification result by time from 1 min to 10 min (line indicates 95% CI).

**Figure 4:**
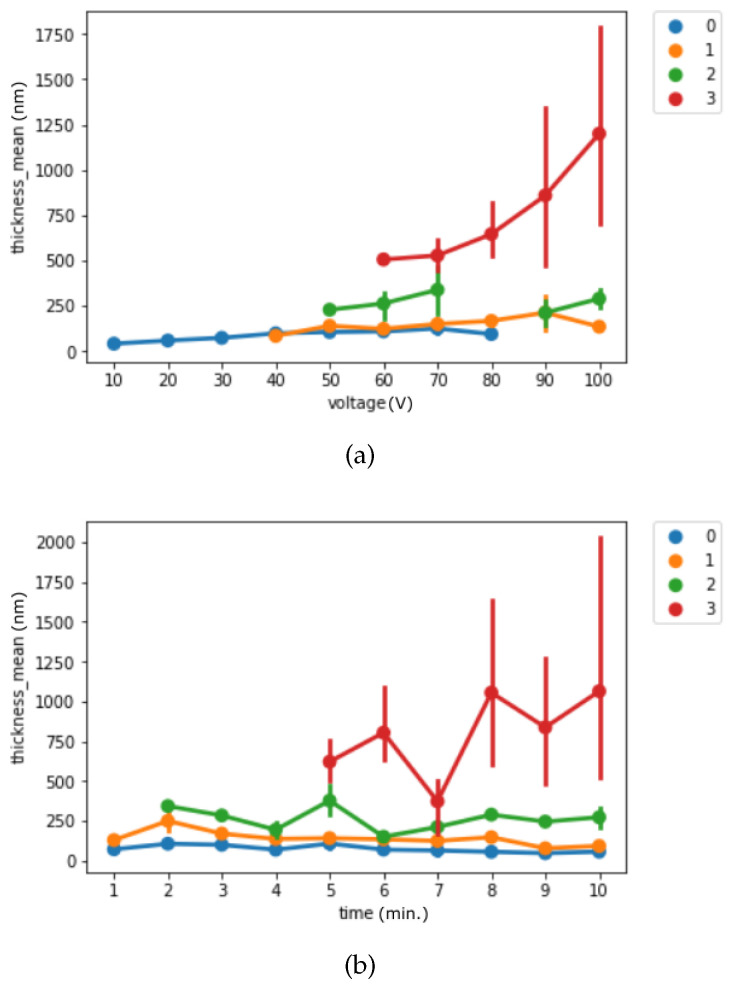
(**a**) Binary classification result by voltage from 10 V to 100 V (**b**) Binary classification result by time from 1 min to 10 min (line indicates 95% CI).

Next, we visualized the thickness by experimental settings and separated them by the labels we used for classification to understand the thickness difference among each classification group. Figure 3 presents the binary classification and Figure 4 presents the multi-class classification. For the binary classification, we can observe that surfaces without pore structure are thinner than the surfaces with pore structure.

### 4.3. Classification Results

#### 4.3.1. Prediction on Binary Classification

Although AUC is our core measure, we report accuracy, precision, and recall measures together. As shown in Table 3, the AUC scores ranged between 0.93 and 1.00. A high AUC value greater than 0.8 denotes a reasonably good prediction rate and all the scores are above this value [37]. Compared to the other models, RF and GBT produced superior AUC scores. As mentioned in Section 3.2, the two algorithms are known as ensemble learning methods utilizing decision trees. The superiority of these models could be attributed to the following reasons. RF consists of a large number of individual decision trees that operated as an ensemble, in other words, the wisdom of crowds [27]. GBT model’s key learning is from the previous mistakes [42]. It relies on the assumption that calculating the best next model combining with previous models, minimizes the overall prediction error that will lead to better performance. KNN is known to have advance in short execution time; however, it has shown to have the lowest accuracy for other classification problems [34].

#### 4.3.2. Multiclass Classification

Table 4 shows the evaluation metrics for multiclass classification. The macro precision scores ranged from 0.42 to 0.73 and macro recall scores ranged from 0.53 to 0.74. For both cases DecTree has relatively higher scores than the other models. Considering accuracy, DecTree, Bagging, and GBT had relatively higher accuracy than the other models. LogReg, SVM, and KNN had relatively lower accuracy. The ensemble methods were superior as binary classification. DecTree is widely known to be powerful for classification because the tree tries to infer a split at each node.

## 5. Discussion

There are several implications that we can achieve from the visualization (see Figure 2). First, we can detect if there is a correlation or trend among the features. For example, one can see that there is a positive increase as time and voltage increase. Second, we can also see that there are similar thicknesses generated from different settings. For example, the size of the circle is similar with the following settings: time (6 min) and voltage (70 V); time(10 min) and voltage(90 V). Even for the same thickness, there are certain levels of time or voltages that are preferred because of stability and also safety issues during the experiment. Third, if you want to determine the specific thickness, there is a minimal level that should be required. If we could predict that then several experiments could be saved. For example, to achieve thickness over 1000, a large amount of experiment settings on the left and lower sides was not needed. Therefore, we believe that if we have a large amount of experimental data, it could be narrowed into meaningful experimental setups.

Prediction scores for binary classification in this study show that our model is capable of predicting the presence of pores using the experimental levels of time and voltages. Researchers can predict whether pores will exist with the experimental settings. With the multiclass classification the evaluation scores were relatively lower. However, even with unbalanced and small sample size to predict four levels, over 0.7 for macro recall and precision shows the potential to move forward with the approach. Higher micro scores can be also interpreted; that is, certain levels have higher prediction scores. To obtain a reliable classification model even for small data sets, Beleites et al. recommended at least 75 to 100 samples per class to achieve a reasonable precision [43].

Overall, we can see that ensemble methods are superior for the binary classification problem. RF and GBT had a score of 1 for AUC. Ensemble methods are learning models based on the intuition that combining the opinions of multiple learners lead to better performance. RF uses a fully grown decision trees that have low bias, which are prone to overfitting. The idea is to resample the data multiple times and train a new classifier each time independently. In contrast, GBT adds a classifier each time, so that the next classifier is trained to improve the previous one. Both cases have shown good performance because they combine multiple individual models and show superior prediction power [44]. For multiclass classification, algorithms based on decision trees had relatively higher accuracy values. In our case, we had an imbalanced data set. A dataset is called imbalanced when the number of samples is different between each class. In this case the accuracy decreases for the minority classes. Decision tree algorithms are robust in these cases; therefore, we believe the DecTree algorithm and the ensembles (i.e., RF, bagging, GBT) also show relatively good performance. The KNN algorithm show the lowest performance for both binary and multiclass classifications. However, the score of 0.97 still shows a high performance for the binary classification problem. KNN is a robust and nonparametric machine learning algorithm. However, the algorithm depends greatly on the distances between points. If the classes overlap, the performance will decrease and we believe this may be the reason for relatively low performance [45]. Considering that this was the first study that comprised 100 samples and applied machine learning techniques for prediction, if we increase the sample size and get a better balanced dataset, we will get a more stable prediction on comparing the algorithms.

## 6. Conclusions

The present work demonstrates that the well-aligned formation of *TiO*_2_ nanostructures can be created in an ethylene glycol-based electrolyte by one-step anodization performed over various anodization voltages at anodization time ranging from 1 min to 10 min. Anodization time and applied voltage have a great impact on the nanostructures of *TiO*_2_ and regularity of pore arrangement. With increasing applied voltage, the growth of oxide thickness increases. In addition, it was found that the oxide thickness changes its thickness in time with increasing tendency and decreasing tendency at specific anodization time and voltage. The highest values of oxide thickness and the regularity of the pore arrangement are observed at 100 V and 10 min as well. These consequences are described in terms of the blocking effect on the *TiO*_2_ surface caused by developed hydrolysis. On the above-obtained results, the ability to control the growth of *TiO*_2_ is an important factor towards a targeted development of *Ti* nanostructure because it is expected that a variety of nano-geometry industry exists.

Therefore, we also applied machine learning to test the feasibility of predicting the surface aspects by the experimental settings, voltage and duration. We have observed that predicting the binary classification had higher accuracy than predicting the multiclass classification. However, the size of the dataset is relatively small than usual machine learning experiments. This is an innate limitation when applying machine learning techniques to experimental data for material science. We believe we have demonstrated classification capability to predict certain aspects from experimental results, which can help identify less useful experimental settings and properly use the cost and time to focus on specific experiments to achieve accurate results.

## Figures and Tables

**Table 1:** Definition of four classes of pore structure and sample images.

Binary Class	Without Pore		With Pore	
**Multiclass**	**Class 0**	**Class 1**	**Class 2**	**Class 3**
Definition	Only layer	Layer with roughness	Unstable pore	Uniform pore
Layer Creation	◯	◯	◯	◯
Layer with Roughness		◯	◯	◯
Pore Creation			◯	◯
Pore with Certain Height				◯
Sample Image	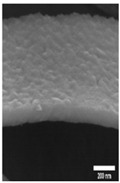	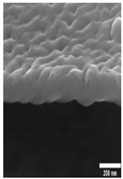	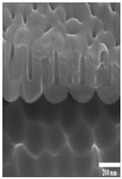	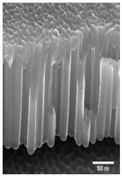
Thickness(nm)	76.48±32.90	147.89±53.86	267.65±84.99	819.60±544.35
# of samples	50	14	13	23

**Table 2:** Cross-sectional view of field emission scanning electron microscope (FE-SEM) images of *TiO*_2_ films formed in the anodization performed at 10–100 V at the anodization time of 1–10 min.

	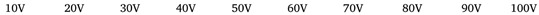
A (1 min.)	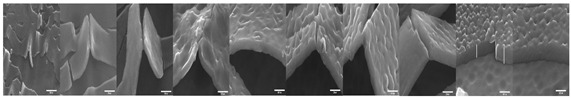
B (2 min.)	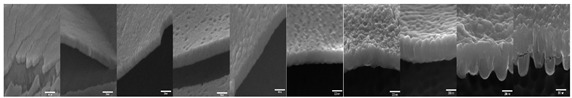
C (3 min.)	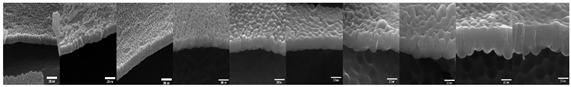
D (4 min.)	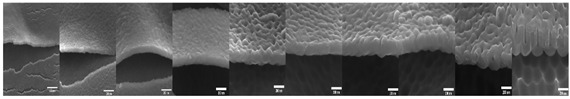
E (5 min.)	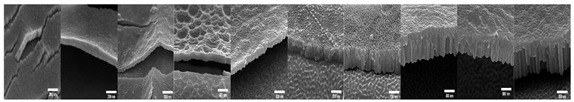
F (6 min.)	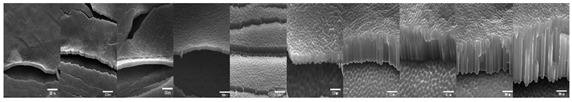
G (7 min.)	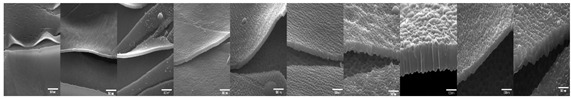
H (8 min.)	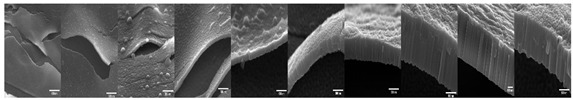
I (9 min.)	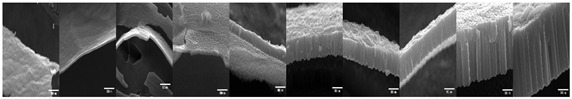
J (10 min.)	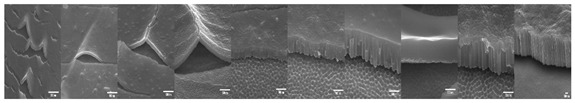

**Table 3:** Comparison of evaluation metrics for classification algorithm on binary classification.

Algorithms	AUC	Accuracy	Precision	Recall
LogReg	0.98	0.90	0.88	0.90
NB	0.99	0.91	0.92	0.88
KNN	0.93	0.88	0.87	0.86
SVM	0.97	0.87	0.93	0.75
DecTree	0.91	0.92	0.94	0.88
RF	1.00	0.91	0.94	0.85
Bagging	0.97	0.90	0.96	0.90
GBT	1.00	0.93	0.94	0.90

**Table 4:** Comparison of evaluation metrics for classification algorithm on multiclass classification.

Algorithms	Accuracy	Micro Precision	Micro Recall	Macro Precision	Macro Recall
LogReg	0.74	0.74	0.74	0.42	0.53
NB	0.78	0.78	0.78	0.60	0.65
KNN	0.70	0.70	0.70	0.52	0.57
SVM	0.74	0.74	0.75	0.55	0.57
DecTree	0.82	0.84	0.84	0.73	0.74
RF	0.79	0.79	0.79	0.66	0.65
Bagging	0.81	0.80	0.77	0.70	0.65
GBT	0.80	0.80	0.80	0.63	0.69

## Data Availability

Data Sharing Not Applicable.
